# RE: Migration of endotacker into the bladder 7 years after laparoscopic retroperitoneal burch application

**DOI:** 10.1590/S1677-5538.IBJU.2015.0273

**Published:** 2015

**Authors:** Ahmet Salvarci, Yunus Agrali

**Affiliations:** Department of Urology, Konya Hospital, Semsi Tebrizi Mh. Serafettin Caddesi, Konya, Turkey; Department of General Surgery, Konya Hospital, Semsi Tebrizi Mh. Serafettin Caddesi, Konya, Turkey

*To the editor,*

I read with interest the recent case reported by Salvarci et al in which the complication of a migrated intravesical endotacker is reported 7 years after a laparoscopic colposuspension (1).

The authors state that during their investigation of retrospective laparoscopic Burch complications they did not reveal any endotacker migration into the bladder similar to the case described.

In 2014 I published a case of migrated intravesical staples which had occurred following a laparoscopic Burch Colposuspension 10 years earlier (2). Similar to the case described by Salvarci et al, our patient had also complained of recurrent urinary tract infections following a laparoscopic colposuspension. In 2002, Washington reported a case of migrated intravesical staples that had occurred only 4 years following laparoscopic colposuspension (3).

Therefore although migrated intravesical tackers remain rare they have been widely reported and can occur between 4 to 10 years post operatively.

*Michael S. Floyd Jr., MCh FRCS (Urol)* *Southport & Ormskirk Hospital NHS Trust,* *Town Lane, Kew,* *Southport, Merseyside,* *PR8 6PN, United Kingdom* *E-mail: nilbury@gmail.com*
